# Characterization and Mechanical Proprieties of New TiMo Alloys Used for Medical Applications

**DOI:** 10.3390/ma12182973

**Published:** 2019-09-13

**Authors:** Andrei Victor Sandu, Madalina Simona Baltatu, Marcin Nabialek, Adriana Savin, Petrica Vizureanu

**Affiliations:** 1 Faculty of Materials Science and Engineering, “Gheorghe Asachi” Technical University of Iasi, 700050 Iasi, Romania; simona.baltatu@tuiasi.ro; 2 Romanian Inventors Forum, Iasi 700089, Romania; 3 Institute of Physics, Faculty of Production Engineering and Materials Technology, Czestochowa University of Technology, 42-200 Częstochowa, Poland; nmarcell@wp.pl; 4 National Institute of Research and Development for Technical Physics 47 Mangeron Boulevard, Iasi 700050, Romania; asavin@phys-iasi.ro

**Keywords:** titanium alloy, biomaterial, TiMoZrTa, TiMoSi, mechanical properties, low elasticity modulus, corrosion

## Abstract

Ti-based alloys are accessible for use in the human body due to their good mechanical properties, corrosion resistance, and biocompatibilities. These main properties of alloys are important criteria for choosing biomedical implants for human bones or for other kinds of applications in general medicine. This paper presents a comparison of four new Ti-based alloys desired to satisfy various requirements for biomedical implants. The materials were prepared with recipes for two new system alloys, TiMoZrTa (TMZT) and TiMoSi (TMS), alloys with nontoxic elements. The presented research contains microstructure images, indentation tests, Vickers hardness, XRD, and corrosion resistance, showing better characteristics than most commercial products used as implants (Young’s modulus closer to the human bone).

## 1. Introduction

At present, the market request for biomaterials is very high. The uses of biomaterials in medical applications includes several areas: Orthopedics, cardiovascular surgery, ophthalmology, dentistry, urology, aesthetic surgery, neurology, suture material for wound healing, and controlled release drug delivery systems. Due to this, it is very important to develop new materials with enhanced properties, biocompatibility, and long-term viability as an implant material. Fundamental requirements are that an implant should have biomechanical properties (stiffness, strength, fracture toughness, wear resistance, fatigue strength, corrosion resistance) and biomedical properties (toxicity, surface state, osseointegration) [1,2,3].

The usual materials currently found in medical applications are classical metallic alloys: Titanium alloys (TiAlV), cobalt alloys (CoCrMo), and stainless steels.

The microstructure, as well as the properties of the titanium alloys, differs depending on the amount of α or β stabilizing elements added to the titanium alloys. Ti-based are grouped into α-type, (α + β)-type, and β-type alloys. Alloying the pure titanium with β-elements leads to the widening of the β-phase domain, as well as the improvement of the mechanical properties proven in the literature [4,5,6,7].

Mo, V, Nb, and Ta are β-stabilizers elements that decrease the temperature of allotropic transformation and neutrals, such as Zr. Commercial pure titanium and Ti_6_Al_4_V alloy were the first titanium-based materials for commercial use. In recent years, further studies have indicated that vanadium, used to stabilize the α-phase, may produce harmful oxides for the human body [8,9].

The authors of [10,11] concluded that Ti, B, Mg, Si, P, Ca, Sr, Zr, Nb, Mo, Pd, In, Sn, Ta, Pt, and Au are biocompatible elements, while harmful elements include Be, Al, V, Cr, Mn, Fe, Co, Ni, Cu, Zn, and Ag [10,11]. 

One of the most important features of an implant is that it comes in contact with the living tissues of the body, thus creating an interface between them. The phenomena that occur at this interface are of great interest because they ultimately determine the success or failure of the implant, both in terms of immediate reaction and long-term response. The biological response between the implant and the host tissue is largely dependent on the place of implantation and the surface properties of the implant. The role of biomaterials is to come into contact with a biological system. When a biomaterial is placed in the human body in the form of a medical device, human tissues react to its implantation in different ways, depending on the type of biomaterial used. The mechanism of cell attachment to the implant depends on the tissue response to the surface of the alloy.

Most recently, researchers have aimed to improve properties by introducing nontoxic elements, for enhancing surface characteristics, mechanical properties, corrosion resistance, biocompatibility and developing alloys with low modulus β or near β Ti alloys, such as Ti–Mo systems, Ti–36Nb–2Ta–3Zr, Ti–24Nb–4Zr–7.8Sn [2,12], etc.

Titanium is a nontoxic element, even in larger quantities; some studies have demonstrated the influence of ingestion by humans of up to 0.8 mg of titanium daily, proving that titanium was eliminated without being digested/assimilated. Its uses in medical applications were due to good interaction with the host bone, the titanium implants not being rejected by the body, and having a high corrosion resistance [13,14,15].

Molybdenum is an element with a lower degree of toxicity compared to Co, Ni, and Cr, and moreover, is a β-stabilizing element. Field studies have highlighted that titanium alloyed with molybdenum in varying percentages between 15%–20% can decrease the modulus of elasticity leading to adequate mechanical properties.

Silicon reduces ductility, improves creep and high-temperature resistance, and increases corrosion resistance.

Zirconium is used in medical applications due to its low modulus of elasticity, high corrosion resistance, and high biocompatibility with human tissue [13,14,15]. 

Tantalum is considered biocompatible, being a β-stabilizing element that influences the value of the modulus of elasticity [13,14,15].

Silicon is an element found in the human bone and is considered biocompatible. It is also a β-stabilizing element that influences the decrease of the modulus of elasticity. 

Despite these facts, Mo, Zr, Ta, and Si were selected for designing a new ß-type of Ti-based alloys (Table 1), systems like TMZT (TiMoZrTa) and TMS (TiMoSi) with lower elastic modulus, corrosion resistance, and good biocompatibility.

This article presents new recipes for Ti-Mo system alloys, with good characteristics and using nontoxic elements, which can be used in medical applications.

## 2. Experimental

### Materials and Preparation

In order to obtain titanium alloys, it was chosen to use a vacuum arc melting plant, produced in house by Institute of Physics, Faculty of Production Engineering and Materials Technology, Czestochowa University of Technology, Poland, and we used raw materials with a high purity: Ti—99.8%, Mo—99.7%, Zr—99.2%, Ta—99.5%, and Si—99.2%.

**Figure 1:**
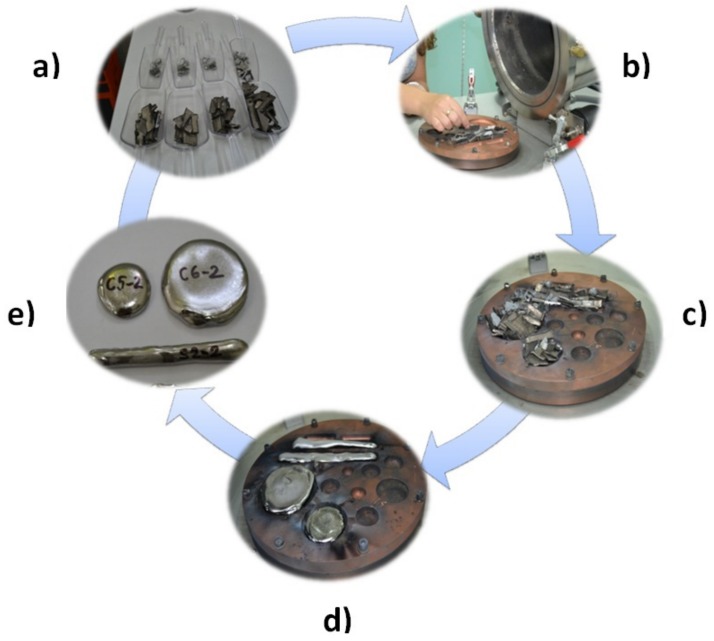
Stages of development of the alloys: (**a**) weighing of raw materials and gravimetric dosing; (**b**,**c**) loading of the raw material; (**d**,**e**) TiMoZrTa (TMZT) semi-products obtained after solidification.

The advantages of this equipment are as follows: High melting temperatures, the use of vacuum or protective environment, uniform composition by repetitive melting, and the possibility of melting elements at different melting points (Figure 1).

The chemical composition of the alloys was analyzed using a scanning electron microscope (SEM) VEGA II LSH manufactured by the TESCAN Co., Brno, the Czech Republic, coupled with an EDX QUANTAX QX2 detector manufactured by the BRUKER/ROENTEC Co., Berlin, Germany. The chemical analyses (EDX) of the alloys studied were performed at many different points for a precise determination, with a uniform distribution of elements and a homogeneous alloy. The chemical composition is shown in Table 1.

The phase analysis was carried out using a Panalytical X’Pert Pro MPD diffractometer, by Malvern Panalytical a Spectris company, Almelo, The Netherlands. The parameters used for sample analysis were: Range of angle θ–2θ between 20° and 80°; continuous scanning. In order to determine the constituent phases, by X-ray diffraction, from the Ti-based alloys developed, samples with size of 10 mm × 10 mm × 5 mm were cut and used after they had been polished.

A Wilson Wolpert universal hardness tester 751 N model, by Wilson Instruments an Instron Company, Heerlen, The Netherlands, was used for the Vickers hardness measurements, with a load of 9807 N during 12 s. The hardness measurements were performed on samples obtained from Ti-based alloys with dimensions 10 mm × 10 mm × 3 mm, the surface of the samples being prepared by grinding on abrasive paper. Experimental tests consisted of three determinations in different areas on the surface of each sample, using a 9.81 N pressure force and a 12 second measurement time.

Elastic modulus was measured using a CETR UMT-2 Tribometer, by Bruker, Campbell CA, USA. For micro-scratch analysis, a constant load method was used with a load of 5N on a distance of 4 mm, for a single determination. For indentation tests, samples of Ti-based alloys obtained were cut with a dimension of 17 mm × 5 mm × 5 mm, and the surface of the samples was prepared by grinding with high-grain silicon carbide abrasive paper and polished with alumina suspension until obtaining metallic gloss and removal of surface roughness. The investigated samples were fixed to a flat surface of the test device with screws and clamps. Testing was carried out under dry conditions. A Rockwell diamond-type penetrator was used, having an incisor cone with an angle of 120°, applying a force of 5 N.

To determine the elastic modulus as accurately as possible, three determinations were performed for each alloy. From the mean values, using UMT 2 software, provided with the CETR UMT-2 Tribometer, the indentation curves (depth vs. force) of TiMoSi alloys were plotted using the ViEWER program.

In order to obtain the electrochemical parameters for characterizing the corrosion resistance of elaborated Ti-based alloys, the electrochemical tests of linear polarization were used. This method can directly and quantitatively determine the corrosion rate. To determine the corrosion potential of Ti-based alloys developed, samples of 5 mm × 5 mm × 5 mm were used. Prior to being subjected to the corrosion potential determination, the samples were cut and ground to remove impurities and the Ti oxide film that formed on the surface of the alloy, then they were embedded in Teflon.

The electrical component of an electrochemical cell consists of a voltmeter, measuring the potential of the product. A reference electrode was a saturated calomel electrode in a potassium chloride solution whose potential is reproducible and has a value of 242 mV at a temperature of 25 °C. Additionally, a platinum electrode was used as an auxiliary electrode. Data processing was done with the Volta Master 4 -Electrochemical Software, by Radiometer Analytical SAS, Villeurbanne, France, then imported and processed using experimental data processing software.

## 3. Results and Discussions

**Figure 2:**
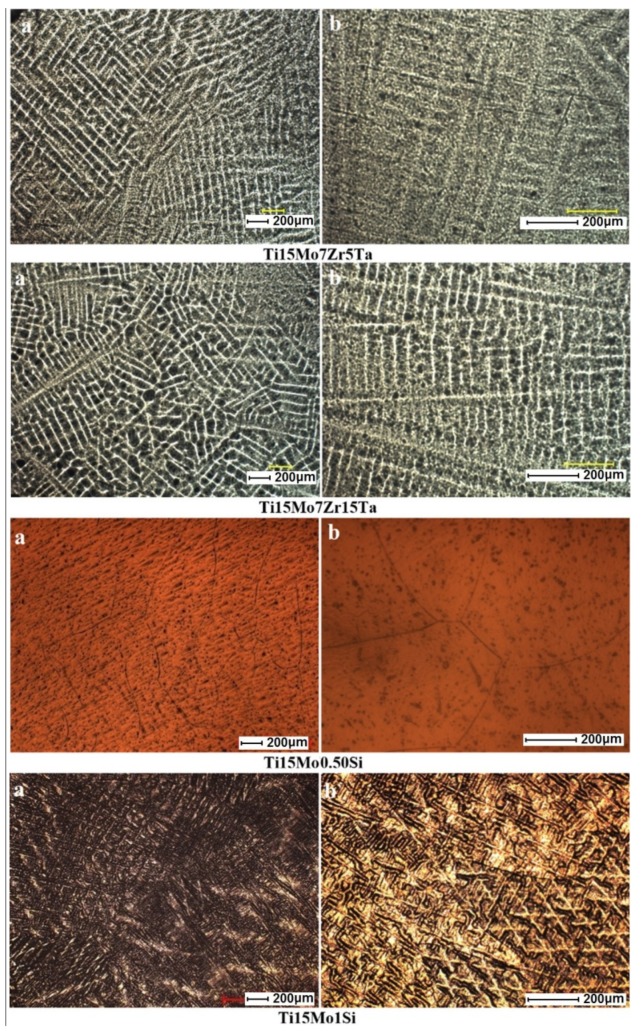
Optical microstructure images of titanium alloys Ti_15_Mo_7_Zr_5_Ta, Ti_15_Mo_7_Zr_15_Ta, Ti_15_Mo_0.5_Si, and Ti_15_Mo_1_Si at various magnifications: (**a**) 50×, (**b**) 100×.

Figure 2 shows the structure of Ti-based alloys with titanium alloy grain specifics. Images obtained by optical microscopy for titanium alloys have a dendrite structure for TiMoZrTa alloy systems. TiMoSi alloy systems highlight the formation of β-type equiaxed grains, having different dimensions. The acicular and coarse structures are specific to β-alloys. With TiMoSi, a β structure of cube with a centered volume is visible.

The variation of the α, α + β, and β phases consists in the differences in the chemical composition of the constituents. The high percentage of β (Mo, Ta, and Si) stabilizing elements led to the formation of a β-type structure, highlighted very well in the Ti-based alloys obtained. Zirconium contribution in concentrations below 10% contributes to the refining of the microstructure, thus allowing the formation of a homogeneous and uniformly distributed structure. Therefore, elements with percentages of tantalum (5%–15%), combined with a molybdenum concentration of 15%, contribute to the formation of phase β.

Optical microscopy (Figure 2) reveals a uniform biphasic structure, consisting of a high proportion of solid β solutions, in which the dendrite lamellar structures of the orthorhombic martensite α″ are present. Orthorhombic martensite occurs frequently in titanium alloys containing β-stabilizers of the transition metals category, including molybdenum and tantalum. In the present case, the presence of the α″ phase is due to the decomposition of the β phase during the cooling. Regarding the Ti_15_Mo_0.5_Si, the small dark spots are due to pitting corrosion after the chemical etching of the polished surface and do not represent any inclusion or other phases.

The hardness measurements highlight the resistance to the penetration action of an external body and provide information on the behavior of the studied materials. In this way, we can analyze the Ti-based alloys developed for the purpose of fitting them into the specific medical application. The hardness measurements made on the titanium alloys, developed by the Vickers method, are a general method for determining the hardness of metallic materials, commonly used in biomaterial testing.

Some of the mechanical properties of Ti-based alloys developed are presented in Table 2 (hardness and Young’s modulus), compared with two other samples: Commercial pure titanium and Ti_6_Al_4_V. The values of the elasticity modulus of the titanium alloys developed, resulting from the indentation test, are highlighted in same table.

According to the literature [15,16,17,18,19], classical biomaterial alloys like stainless steel and CoCrMo alloys have hardness values between 155 HV and 601 HV. The titanium alloys obtained comprise values ranging from 188.52 to 390.88. Titanium alloys have values approximate to the Ti_6_Al_4_V alloy (349 HV), which is mostly used in implantology [15,16,17,18,19].

The elasticity module is a very important criterion underlying the choice of metallic materials used in orthopedics and should be as close as possible to human bone (17–30 GPa) to avoid the stress-shielding effect [1,2,3]. 

The Ti-based alloys obtained have values ranging from 19.82–69.02 GPa for the modulus of elasticity, measured by indentation tests. The lowest value is the Ti_15_Mo_0.5_Si alloy (19.82 GPa), and the highest value are the Ti_15_Mo_7_Zr_5_Ta alloys (69.02 GPa). The low elasticity modulus of the investigated alloys is due to the presence of β-stabilizing elements such as Mo, Ta, and Si. According to Table 2, it can be seen that adding elements like Zr and Ta can increase values of modulus of elasticity with approximately 40 GPa. On the other hand, increasing the percentage of Ta from 5% to 15% contributed to a decrease in the modulus of elasticity for TMZT alloys.

As we can see from the data in Table 2, the highest Young’s modulus (69 GPa) is visible in the Ti_15_Mo_7_Zr_5_Ta systems, due to the presence of α″ phase (martensite). Ti_15_Mo_0.5_Si presents the lowest Young’s modulus (20 GPa), where on the microstructure, the β boundaries are clearly visible (Figure 2b). These are confirmed also by the literature, that the β phase presents better mechanical properties. Taking into consideration other metallic biomaterials—CoCrMo alloys (210–253 GPa) and stainless steels (190–210 GPa)—the investigated Ti-based alloys exhibited much lower values and closer values to that of the human bone (17–30 GPa). The alloys developed showed a significant improvement for mechanical properties even for titanium alloys: Ti_6_Al_4_V (100–114 GPa) and C.P. Ti (102–104 GPa). 

From the point of view of elasticity, these alloys fulfilled the most important requirement criteria of metallic biomaterials, the values of the modulus of elasticity being close to that of the human bone.

**Figure 3:**
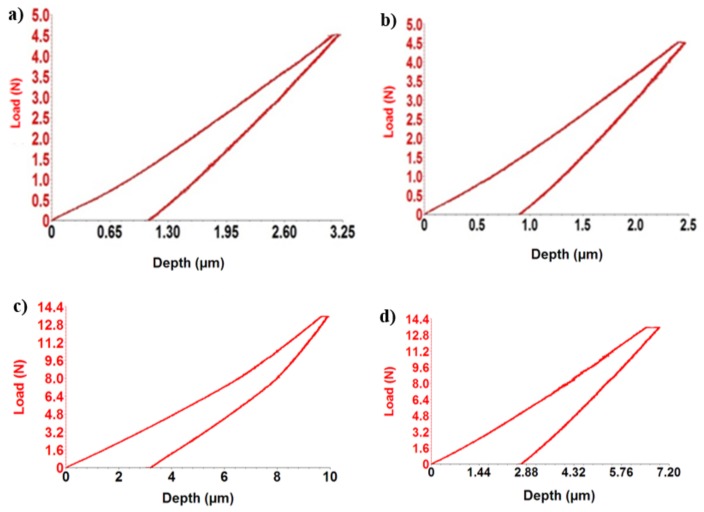
The strength-depth curve of the indentation test for Ti-based alloys developed: (**a**) Ti_15_Mo_7_Zr_5_Ta, (**b**) Ti_15_Mo_7_Zr_15_Ta, (**c**) Ti_15_Mo_0.5_Si, and (**d**) Ti_15_Mo_1_Si.

Figure 3 shows the response of alloys during indentation tests in the form of force–depth dependencies.

**Figure 4:**
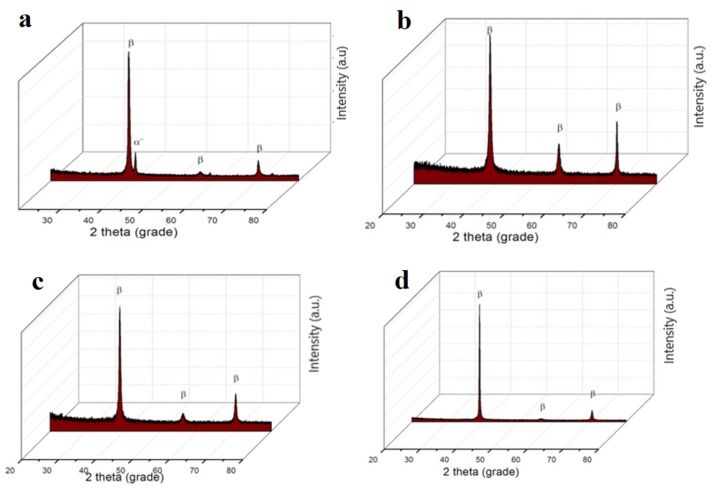
The XRD diffraction patterns of titanium samples: (**a**) Ti_15_Mo_7_Zr_5_Ta, (**b**) Ti_15_Mo_7_Zr_15_Ta, (**c**) Ti_15_Mo_0.5_Si, and (**d**) Ti_15_Mo_1_Si.

The diffractograms obtained (Figure 4) show the β-type structures identified by optical microscopy, taking into account that titanium is an allotropic element, presenting it in different forms: Up to a temperature of 882 °C, having a α-Ti compact hexagonal structure and above 882 °C, β–Ti, having a centered cube structure. In the composition of the investigated alloys, there is a major phase β with a centered cube structure and a secondary phase α″ with an orthorhombic structure (martensite), present only for Ti_15_Mo_7_Zr_5_Ta systems.

Phase β, having a centered cubic structure, is fixed by alloying the titanium with transition elements (Mo, Ta, and Si) crystallized in the orthorhombic system and is formed when the content of stabilizing elements phase β decomposed during cooling [20,21,22,23].

The corrosion of biomaterials is determined by the aggressive corrosive nature of the elements present in body fluids. In order to determine the corrosion potential of titanium alloys developed, it was necessary to investigate them through potentially dynamic and potentiostatic tests in simulated biological environments.

The simplest method of measuring the corrosion rate of a metal involves putting it in contact with the test medium and measuring the amount of material lost by the sample depending on the exposure time.

The polarization resistance method was used to assess the corrosion rate for the Ti-based alloys developed. This method serves to determine the corrosion current and the corrosion potential of the metal or alloy, from the linear polarization curve obtained from relatively small voltages. The corrosion current determined by this method is therefore the current that occurs at the metal/corrosive environment interface when the metal is immersed in the solution and represents the instantaneous corrosion current.

The specific titanium developed samples were introduced into the electrochemical cell, and the selected corrosion medium was Ringer’s solution with the composition: NaCl: 8.6 g/L, KCl: 0.3 g/L, CaCl_2_: 0.48 g/L, in order to investigate their use as potential biomaterials. Measurements were made at 20 °C in naturally aerated solutions. The anodic polarization curves were recorded at a potential sweep rate of 0.5 mV/s over a potential range of ± 200 mV versus the open circuit potential. Corrosion potential (E_corr_ = E (I = 0)), Tafel slopes (βa and βc), polarization resistance (R_p_), instantaneous corrosion density (corrosion), and corrosion rate were evaluated using the VoltaMaster 4 software.

**Figure 5:**
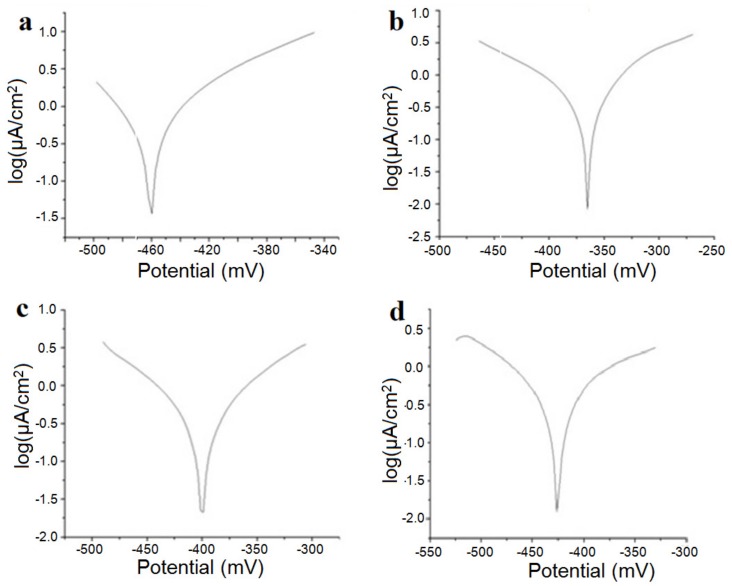
Diagrams of the investigated alloys by corrosion testing in Ringer’s solution of the samples: (**a**) Ti_15_Mo_7_Zr_5_Ta, (**b**) Ti_15_Mo_7_Zr_15_Ta, (**c**) Ti_15_Mo_0.5_Si, and (**d**) Ti_15_Mo_1_Si.

Figure 5 shows the linear polarization curves in semilogarithmic coordinates for the samples studied in the Ringer solution, and in Table 3, we present the instantaneous corrosion parameters in the same physiological environment.

Corrosion potential, E_corr_, measured relative to the potential of the saturated calomel electrode, is the potential at which the oxidation–reduction reactions on the alloy surface are at equilibrium; the rate of the oxidation reaction is equal to the rate of the reduction reaction, and the total current intensity is zero. Increasing the potential to more positive values increases the rate of the oxidation reaction, while the potential shift to negative values is reduced by the oxidation process, and the metal passes. It can be seen that the presence of silicon appears to produce an increase in the corrosion rate.

Polarization resistors have high values, which is reflected in very low corrosion rates. The “corrosive” product of these alloys is mainly titanium oxide, TiO_2_, which is insoluble and adherent to the surface of the alloy. The oxide layer on the surface protects the alloy against the aggressive action of electrolytic media. Considering this, it can be concluded that in the physiological environment, titanium-based alloys are not corroded but actually suffer a passivation process. Under these conditions, the variable corrosion rate is actually passivation velocity.

Particular attention should be given to the Ti15Mo1Si alloy; in these cases, the polarization resistances were small and the corrosion rates were higher than that for the other alloys (almost double). 

The value of molybdenum and tantalum concentrations in the studied alloys are important for corrosion studies. The higher the percentage of tantalum, the better the characteristics of corrosion resistance. Molybdenum has a great influence on the corrosion parameters; thus, for a percentage of 15%, the corrosion resistance is very good [24,25,26,27].

## 4. Conclusions

At the global level, there is continuous concern for the research and development of alloys for medical and biomedical applications. Thus, it is desired to improve both the classic technologies of obtaining implants and the technologies of synthesis of the biomaterials from which they are executed, having as the final aim the promotion of a new generation of multifunctional implants with long-term performance.

Biomaterials are synthetic materials used to replace a part of a living system, or to function in close connection with a living tissue, which is why they must have properties as close to that of the human bone. Young’s modulus of the Ti-based alloys obtained values ranging from 19.82 to 69.02 GPa, which is recommended for alloys used in implantology.

For most of the samples, the process that takes place on the surface is the oxidation of the titanium with the formation of an oxide adherent to the surface, which in fact produces the passive alloy. The corrosion rates (in fact passivation rates) are of the same order of magnitude, relatively small (20–30 m/year), for all alloys with uniform composition.

This paper presents the effect of tantalum addition that is beneficial in decreasing the modulus of elasticity as well as the corrosion rate. The addition of silicon has shown that it can present good properties only in a percentage of less than 0.5.

In conclusion, we can state that the characteristics of titanium alloys depend on the alloying elements, influencing the mechanical properties, and the biocompatibility. Elements like molybdenum, zirconium, tantalum, and silicon alloyed with titanium show good properties; in the future, they can be introduced in different medical applications such as dental and orthopedic implants.

## Figures and Tables

**Table 1:** Chemical composition of alloys after melting obtained by Energy Dispersive X-ray analysis.

Chemical Element	Mo	Si	Zr	Ta	Ti
wt.(%)	13.56	-	6.34	4.64	Balance
	12.56	-	6.99	14.45	Balance
	15.93	0.28	-	-	Balance
	15.19	1.02	-	-	Balance

**Table 2:** Some mechanical properties of titanium samples.

Alloy	Hardness (HV)	Young Modulus (GPa)
Ti_15_Mo_7_Zr_5_Ta	379.21	69.02
Ti_15_Mo_7_Zr_15_Ta	390.88	51.93
Ti_15_Mo_0.5_Si	233.37	19.82
Ti_15_Mo_1_Si	188.52	42.84
C.P. Titanium	182	105
Ti6Al4V	541	119

**Table 3:** The main parameters of the Ringer solution corrosion process.

Alloy	E_corr_ [mV]	β_a_ [mV]	β_c_ [mV]	R_p_ [kΩ/cm^2^]	J_cor_ [µA/cm^2^]	V_cor_ [µm/year]
Ti_15_Mo_7_Zr_5_Ta	−461.20	117.90	−39.50	22.00	1.08	12.67
Ti_15_Mo_7_Zr_15_Ta	−365.40	88.30	−115.00	38.78	0.47	5.49
Ti_15_Mo_0.5_Si	−266.00	200	−192	14.91	2.13	20.59
Ti_15_Mo_1_Si	−309.00	278	−152	8.99	3.96	38.30
